# Quantum interference in the presence of a resonant medium

**DOI:** 10.1038/s41598-017-11694-z

**Published:** 2017-09-13

**Authors:** Dmitry A. Kalashnikov, Elizaveta V. Melik-Gaykazyan, Alexey A. Kalachev, Ye Feng Yu, Arseniy I. Kuznetsov, Leonid A. Krivitsky

**Affiliations:** 1 Data Storage Institute, Agency for Science, Technology and Research (A*STAR), 138634 Singapore, Singapore; 2 Faculty of Physics, M. V. Lomonosov Moscow State University, 119991 Moscow, Russia; 3 Zavoisky Physical-Technical Institute of the Russian Academy of Sciences, 420029 Kazan, Russia

## Abstract

Interaction of light with media often occurs with a femtosecond response time. Its measurement by conventional techniques requires the use of femtosecond lasers and sophisticated time-gated optical detection. Here we demonstrate that by exploiting quantum interference of entangled photons it is possible to measure the dephasing time of a resonant media on the femtosecond time scale (down to 100 fs) using accessible continuous wave laser and single-photon counting. We insert a sample in the Hong-Ou-Mandel interferometer and observe the modification of the two-photon interference pattern, which is driven by the coherent response of the medium, determined by the dephasing time. The dephasing time is then inferred from the observed pattern. This effect is distinctively different from the basic effect of spectral filtering, which was studied in earlier works. In addition to its ease of use, our technique does not require compensation of group velocity dispersion and does not induce photo-damage of the samples. Our technique will be useful for characterization of ultrafast phase relaxation processes in material science, chemistry, and biology.

## Introduction

Upon coherent light excitation of a media, its evolution is basically described by two processes: the nonequilibrium population decay and dephasing (or disorientation) of the induced dipole moments. The latter process is determined by phase relaxation (dephasing) time *T*_2_^1^. Accurate measurement of *T*_2_ is essential for characterization of a number of processes including, atomic collisions, molecular vibrations, studies of surface states and others^2–7^.

For many processes, *T*_2_ lies within the femtosecond time scale and is typically measured by the methods of time-resolved spectroscopy^1, 8, 9^. These techniques are universally applied to both optically thin and thick samples, as it allows accounting for an additional spectral broadening of resonant absorption lines^10^. The techniques are capable of resolving the homogenous dephasing time ${T}_{2}^{^{\prime} }$ from the total dephasing time *T*_2_, which accounts the additional broadening due to inhomogeneity of a surrounding medium $1/{T}_{2}=1/{T}_{2}^{\ast }+1/{T}_{2}^{^{\prime} }$, where ${T}_{2}^{\ast }$ is inhomogeneous life-time^9^. The homogenous dephasing time is limited by the energy relaxation time *T*_1_, $1/{T}_{2}^{^{\prime} }=1/2{T}_{1}+1/{T}_{2}^{pure}$, where ${T}_{2}^{pure}$ corresponds to the pure dephasing processes e.g. spectral diffusion (*T*_1_ is often significantly larger than ${T}_{2}^{pure}$)^1^. However, implementation of the time-resolved spectroscopy faces a number of practical challenges. First, it requires the use of a femtosecond laser system with pulse duration significantly less than the *T*_2_. Second, signal detection with adequate temporal resolution requires the use of nonlinear wave-mixing processes. Moreover, femtosecond pulses have an inherently high peak power, and one has to be careful not to damage and/or modify the sample under study. At the same time, dephasing time *T*_2_ reflects itself in the coherent transient phenomena, which in principle can be measured with linear methods. For example, free-induction decay was measured using interferometric methods with broadband incoherent light^11–13^. However, the limitation of that method is that it still suffers from group velocity dispersion.

Here we report on a new approach which allows measurement of the dephasing time *T*_2_, which in general case corresponds to an inhomogeneously broadened optical transition, with femtosecond time resolution without the need of a femtosecond laser and a sophisticated detection system. We exploit the unique properties of quantum entanglement, which have already gained momentum in addressing a variety of practical applications, including secure communication^14–17^, metrology^18, 19^ and sensing^20–22^.

We generate entangled photons via spontaneous parametric down conversion (SPDC)^23^ and build the two-photon interference setup, known as the Hong, Ou and Mandel (HOM) interferometer. In the HOM interferometer, two indistinguishable photons interfere on a 50/50 beam splitter and then are detected by two single photon photodetectors^24^. Destructive interference of probability amplitudes results in the observation of a pronounced dip in the dependence of coincidences of photocounts on the optical delay referred to as the HOM dip. Conventionally, the HOM interference dip has a symmetric shape, which is defined by the Fourier transform of the power spectrum of entangled photons. The width of the dip is inversely proportional to the coherence time of the photons (typically at the order of few tens to hundreds of femtoseconds)^25–27^. It has been shown that when entangled photons propagate through dispersive media the shape of the HOM dip is not affected by the odd orders of dispersion^28–30^. This effect is referred to as dispersion cancellation and it is central to the quantum optical coherence tomography^31^. A related effect of quantum beatings reveals itself in oscillations in the HOM dip for partially distinguishable photons^32–34^.

It is important to note that in some situations, the shape of HOM interference cannot be described only by the spectral profile. For example, it was shown that the phase acquired by the photons due to even orders of dispersion causes elongation of the dip and its asymmetric oscillations^35–37^. Moreover, modifications of the dip can be caused by any process, which implies an asymmetric spectral phase difference even without modifications to the spectrum^38^.

Here we consider the propagation of entangled photons through a resonant medium introduced in one arm of the HOM interferometer. In this case, the photon can be treated as a small-area pulse, and the shape of HOM dip is now affected not only by the spectral transmission of the medium, but also by a phase acquired due to coherent response of the resonant medium under a single photon excitation^39, 40^. This effect is distinctively different from the basic spectral filtering approach.

Using the theoretical analysis, we infer the dephasing time *T*_2_ from the shape of HOM dip. Due to the fact that coherence length of entangled photons is on the order of tens-hundreds of femtosecond we are able to measure dephasing time on the femtosecond time scale even though entangled states of light are generated using a continuous wave (cw) laser. To the best of our knowledge, the influence of the coherent response of the medium on the shape of the HOM dip was not reported earlier.

## Materials and Methods

### Theoretical considerations for a resonant medium

**Figure 1:**
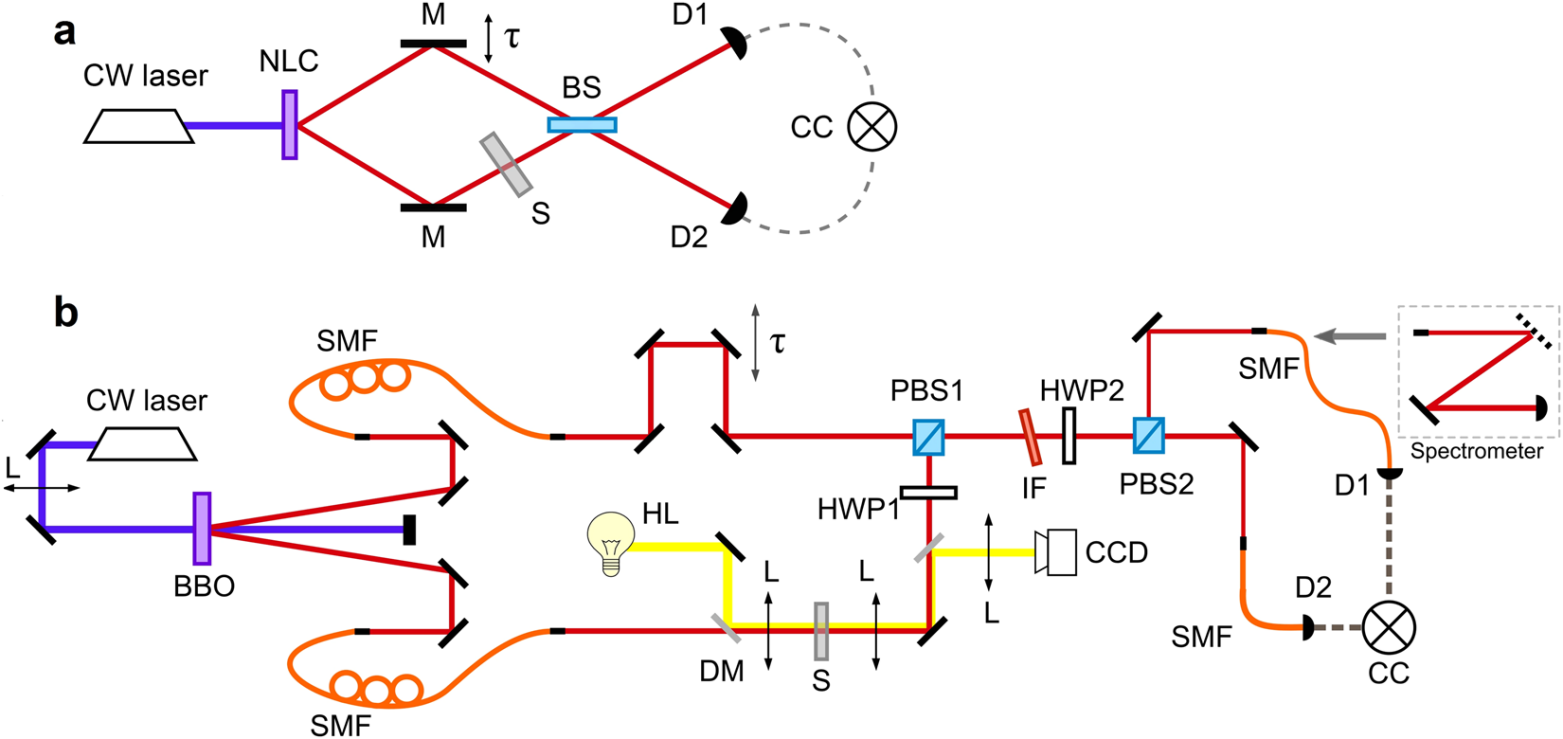
The experimental setup. (**a**) Principal scheme. The pump from a cw-laser passes through a nonlinear crystal (NLC). The SPDC radiation is fed into the interferometer by a set of mirrors (M). Sample (S) is placed in one of the arms of the interferometer. The time delay τ is induced by a mirror on a translation stage. Photons interfere at a 50/50 beamsplitter (BS) and then are detected by avalanche photodetectors (D1 and D2). Signals from detectors are sent to a coincidence counting scheme (CC). (**b**) Experimental setup. A 407 nm cw-laser is focused by a lens (L) onto a BBO crystal cut for type-I SPDC. The SPDC radiation is fed into two single mode fibers (SMF) and then coupled into the interferometer. Sample (S) is placed in one of the arms of the interferometer. An imaging system consisting of two confocal lenses (L) allows observation of a sample on a CCD camera. Time delay τ is introduced by a motorized translation stage. The half-wave plate (HWP1) rotates the polarization at 90 deg and the two beams in the interferometer are combined on a polarization beamsplitter (PBS1). A half-wave plate (HWP2) rotates polarization at 45 deg, and the interference is observed at the output of the PBS2. In HOM interference the photons are detected directly by avalanche photodiodes (D1, D2) connected to a coincidence circuit (CC); for spectral measurements we use a grating spectrometer.

We consider the HOM interferometer with a resonant medium in one of the arms, see Fig. 1a. A biphoton field, produced via SPDC, can be represented as

1 $$|\psi \rangle =\iint d{\omega }_{1}d{\omega }_{2}F({\omega }_{1},{\omega }_{2})|{\omega }_{1}\rangle |{\omega }_{2}\rangle =\iint d{\omega }_{1}d{\omega }_{2}F({\omega }_{1},{\omega }_{2}){a}_{1}^{+}({\omega }_{1}){a}_{2}^{+}({\omega }_{2})|0\rangle ,$$

where *F*(*ω*_1_, *ω*_2_) denotes the biphoton field amplitude and ${a}_{i}^{+}({\omega }_{i})$ denotes the creation operator at frequency *ω*_*i*_, *i* = 1, 2.

In one arm of the interferometer, we introduce an optical delay element with the transmission function *ϕ*(*ω*) (in the absence of losses $|\varphi (\omega )|=1$). In another arm we introduce a resonant medium described by a transfer function *H*(*ω*). Then Eq. 1 can be rewritten as:

2 $$|\psi \rangle =\iint d{\omega }_{1}d{\omega }_{2}F({\omega }_{1},{\omega }_{2}){a}_{1}^{+}({\omega }_{1}){a}_{2}^{+}({\omega }_{2})\varphi ({\omega }_{1})H({\omega }_{2})|0\rangle .$$

After passing through the beamsplitter the state changes to

3 $$\begin{array}{c}|\psi \rangle =\iint d{\omega }_{1}d{\omega }_{2}F({\omega }_{1},{\omega }_{2})[{b}_{2}^{+}({\omega }_{1}){b}_{1}^{+}({\omega }_{2})-{b}_{1}^{+}({\omega }_{1}){b}_{2}^{+}({\omega }_{2})+\\ \quad \,\,\,\,+i{b}_{1}^{+}({\omega }_{1}){b}_{1}^{+}({\omega }_{2})+i{b}_{2}^{+}({\omega }_{1}){b}_{2}^{+}({\omega }_{2})]\varphi ({\omega }_{1})H({\omega }_{2})|0\rangle ,\end{array}$$

where ${b}_{i}^{+}({\omega }_{i})$ is a creation operator at the output of the beamsplitter. The coincidence count rate between the two detectors is determined by the first two terms in Eq. 3. After redefining the integration variables, the relevant part of the state can be written as follows:

4 $$|{\psi }_{c}\rangle =\frac{1}{2}\iint d{\omega }_{1}d{\omega }_{2}[F({\omega }_{1},{\omega }_{2})\varphi ({\omega }_{1})H({\omega }_{2})-F({\omega }_{2},{\omega }_{1})\varphi ({\omega }_{2})H({\omega }_{1})]|{\omega }_{1},{\omega }_{2}\rangle .$$

We can now analyze the dependence of the coincidence count rate on the time delay, assuming that in a typical experiment the time window of the coincidence circuit (typically a few ns) is much larger compared to the coherence time of the field. In this case, the coincidence count rate is given by:

5 $${P}_{c}={\iint d{\omega }_{1}d{\omega }_{2}|\langle {\omega }_{1},{\omega }_{2}|{\psi }_{c}\rangle |}^{2}.$$

Substituting $|{\psi }_{c}\rangle$ and taking into account that in the absence of losses $|\varphi (\omega )|=1$, we can rewrite Eq. 5 in the following way:

6 $$\begin{array}{c}{P}_{c}=\frac{1}{4}\iint d{\omega }_{1}d{\omega }_{2}{|F({\omega }_{1},{\omega }_{2})H({\omega }_{2})|}^{2}+{|F({\omega }_{2},{\omega }_{1})H({\omega }_{1})|}^{2}\\ \quad \,\,-\,2{\rm{\Re }}\{{F}^{\ast }({\omega }_{1},{\omega }_{2})F({\omega }_{2},{\omega }_{1}){H}^{\ast }({\omega }_{2})H({\omega }_{1}){\varphi }^{\ast }({\omega }_{1})\varphi ({\omega }_{2})\},\end{array}$$

where we assume that the time delay is introduced by an optical element without dispersion, and $\varphi (\omega )={e}^{i\omega t}$. For the SPDC pumped by a cw-laser the biphoton field has a strong frequency anticorrelation and the state described by Eq. 1 can be represented as

$$|\psi \rangle =\int d\nu F(\nu ){a}_{1}^{+}({\omega }_{0}-\nu ){a}_{2}^{+}({\omega }_{0}+\nu )|0\rangle ,$$

where *ω*_0_ is the central frequency of the biphoton field, and *v* is detuning from the central frequency. Then, Eqs 2–6 can be rewritten in terms of *ω*_0_ and *v*, and the coincidence count rate is given by

7 $$\begin{array}{c}{P}_{c}(\tau )=\frac{1}{4}\int d\nu {|F(\nu )H({\omega }_{0}-\nu )|}^{2}+{|F(\nu )H({\omega }_{0}+\nu )|}^{2}\\ \quad \quad \,-\,2{\rm{\Re }}\{{|F(\nu )|}^{2}{H}^{\ast }({\omega }_{0}-\nu )H({\omega }_{0}+\nu ){e}^{-i2\nu \tau }\}.\end{array}$$

Equation (7) shows the relationship between shape of the HOM dip and the linear transmission spectrum. We assume that the resonant medium is a two-level system with a Lorentzian line shape

8 $$H({\omega }_{0}+\nu )=\exp [-\frac{ib}{\nu -{\rm{\Omega }}+i/{T}_{2}}],$$

where $b=\alpha L/2{T}_{2}$, *αL* is the optical thickness (*α* is a Bouguer coefficient and *L* is the length of the medium, and *αL* < 1 for an optically thin sample), *T*_2_ is the dephasing time, which in general accounts for an inhomogeneous broadening, and ${\rm{\Omega }}\equiv {\omega }_{res}-{\omega }_{0}$, where *ω*_*res*_ is the resonant frequency.

The effect can be explained from the point of polarization of the medium, induced by a photon, or short-pulse, excitation. This transient polarization persists within the dephasing time *T*_2_ and reshapes the propagating photon. This reshaping reveals itself in the dependence of the coincidence rate in the HOM interferometer. Substituting Eq. 8 in Eq. 7 we can estimate the dephasing time *T*_2_ of the medium from the acquired dependence of a coincidence count rate on time using fitting parameters. First, we numerically study the cases for resonant and non-resonant media, and then perform experiments with two different resonant samples: an Nd:YAG crystal and an array of nanoparticles made of amorphous silicon (see Samples). A simple analytical solution for the case of a single resonant line can be found in the Supplementary Materials.

### Spectral function of the biphoton field

The spectral amplitude of biphoton field *F*(*v*) consists of two components: the phase matching function of the nonlinear crystal *F*_*bp*_(*v*), which determines the width of SPDC spectrum, and the transfer function of the filter, placed in front of the detector Φ(*v*). This yields

9 $$F(\nu )={F}_{bp}(\nu ){\rm{\Phi }}(\nu ).$$

**Figure 2:**
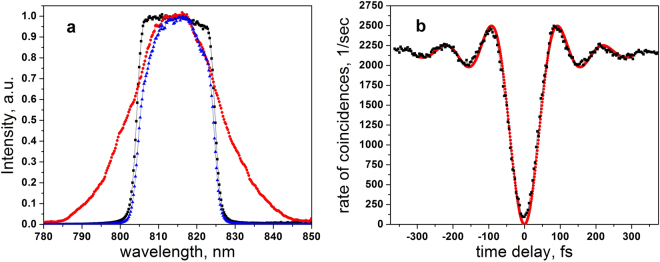
Characterization of the biphoton field. (**a**) Normalized transmission of the interference filter (black) and spectrum of SPDC (red). Their convolution (blue) determines the spectral shape of the biphoton field used in the experiment. (**b**) The HOM dip without samples. Experimental results (black) are fitted by Eq. 7 (red) with measured parameters of the biphoton field.

In type-I frequency-degenerate SPCD, which is used in our experiment, the photons in a pair have the same polarization so that

$${F}_{bp}(\nu )\propto {L}_{c}{\chi }^{(2)}{E}_{pump}{\rm{sinc}}(\frac{{\nu }^{2}D^{\prime\prime} {L}_{c}}{2}),$$

where *L*_*c*_ is the length of the nonlinear crystal, χ^(2)^ is the nonlinear susceptibility, *E*_pump_ is the pump field amplitude, $D^{\prime\prime} ={d}^{2}k/d{\nu }^{2}$ is the group velocity dispersion in the crystal at the frequency of the SPDC field. The SPDC field is restricted by a filter with a trapezoidal shape ${|{\rm{\Phi }}(\nu )|}^{2}$, where an imaginary part of Φ(*v*) is determined by the Hilbert transformation. The spectral function of the biphoton field is derived from Eq. 9 using the measured SPDC spectrum and the transmission curve of the filter, see Fig. 2a. The former has a full width on a half maximum Δλ_1/2_ = 22 nm, and the latter is modeled by a trapezoidal function with a top width of 15.5 nm and side slopes of 3.3 nm.

### Samples

**Figure 3:**
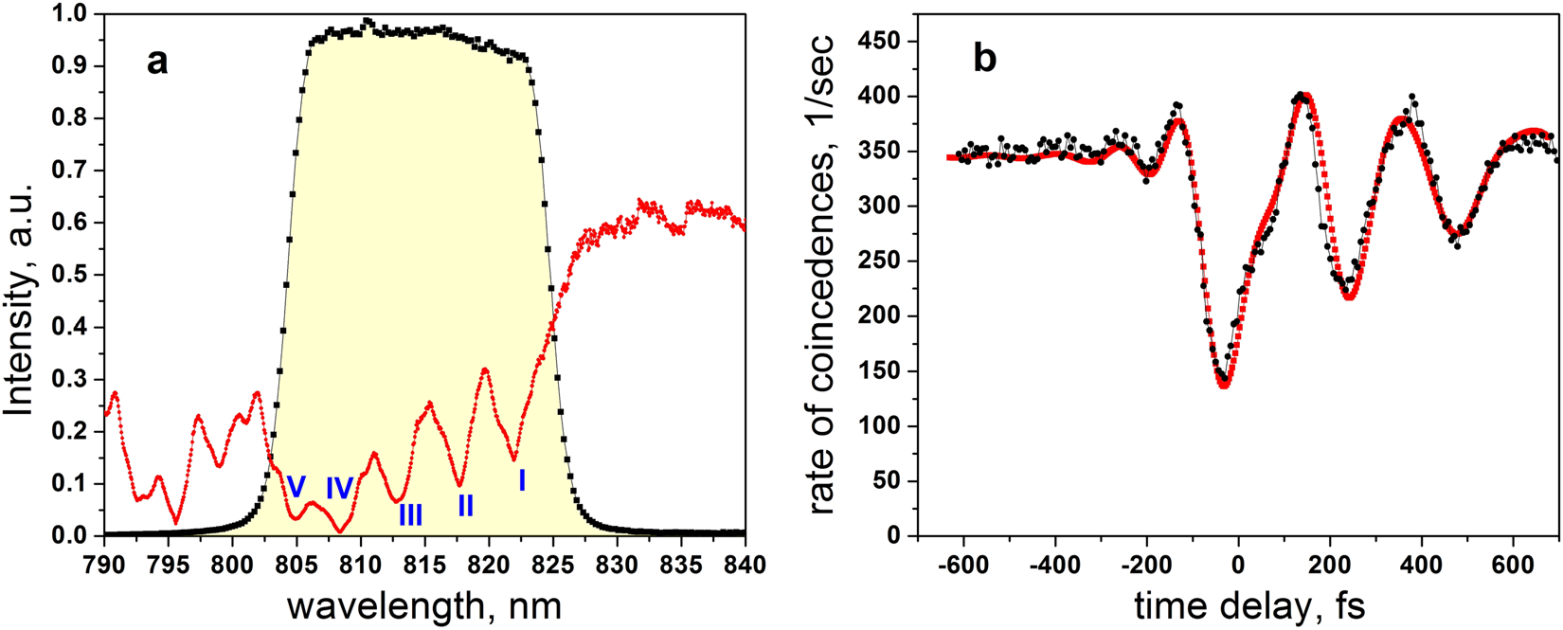
Results for the Nd:YAG crystal. (**a**) The transmission spectrum of the 8 mm long Nd:YAG crystal (red), restricted by an interference filter (black dots and yellow shaded area). The intensity is normalized to the transmission by the filter. Blue roman numbers denote individual absorption lines. (**b**) The HOM dip with the Nd:YAG crystal in one arm of the interferometer. Experimental results (black) are fitted by Eqs 7–9 (red) with the fixed parameters of the biphoton field and the sample, and T_2_ for each of the five lines being a fitting parameter.

The Nd:YAG crystal (Nd concentration 1%; 8 mm length; antireflection coated facets for 800 nm) has a strong absorption line at 808 nm and four satellite lines in the range 804–822 nm, which are within FWHM of the interference filter, see Fig. 3a, where the roman numerals denote the corresponding line numbers. From the obtained spectrum we determine the detuning from the center of the biphoton field for each line: Ω^I^ = 7.5 nm, Ω^II^ = 3.1 nm, Ω^III^ = −1.55 nm, Ω^IV^ = −6 nm, Ω^V^ = −9.9 nm, where the upper index denotes the line number. We estimate the corresponding optical thicknesses to be *αL* ^I^ = 1.95, *αL* ^II^ = 2.35, *αL* ^III^ = 2.9, *αL* ^IV^ = 7.2, *αL* ^V^ = 3.6.

**Figure 4:**
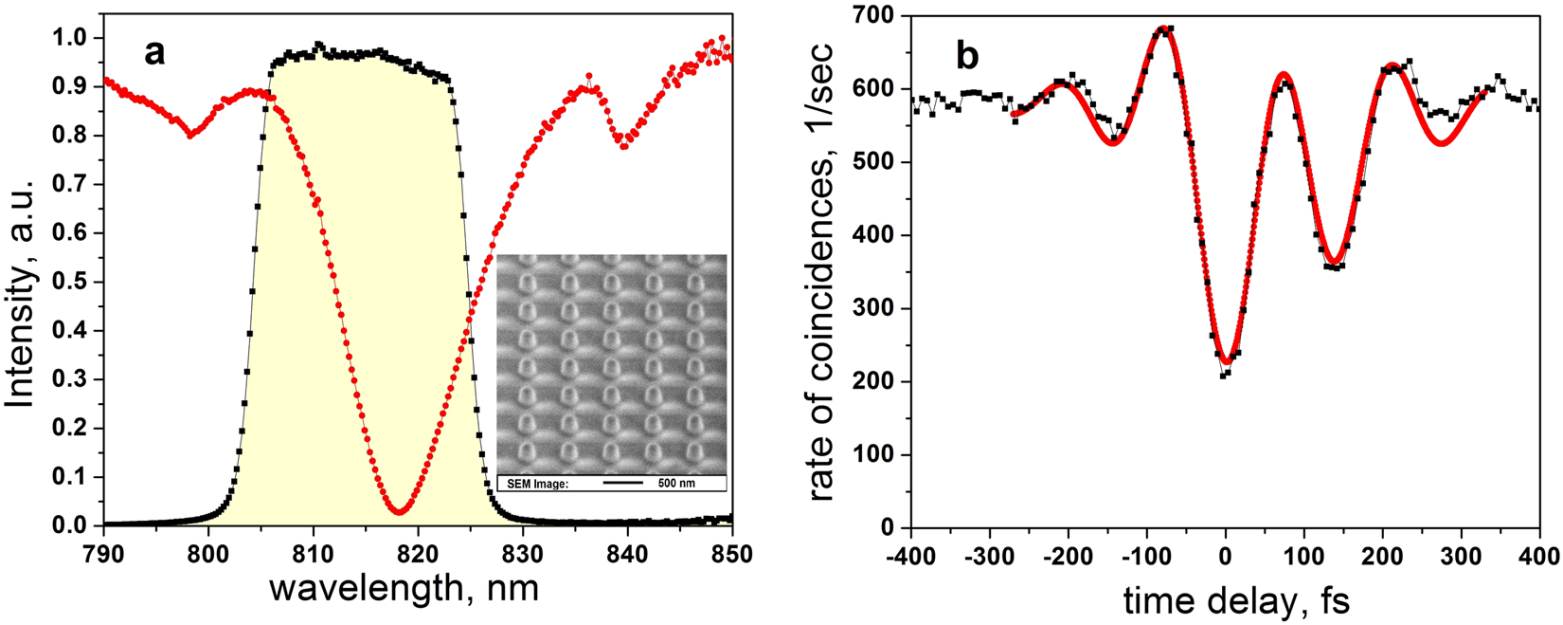
Results for the nanostructure. (**a**) The transmission spectrum of the nanostructure (red), restricted by the interference filter (black dots and yellow shaded area). The intensity is normalized to the transmission of the filter. The inset shows a SEM image of the array of Si nanodiscs with the diameter of 200 nm, disc height of 150 nm and pitch of 550 nm. (**b**) The HOM dip obtained with the nanostructure in one arm of the interferometer. Experimental results (black) are fitted by Eqs 7–9 (red) with the measured parameters of the biphoton field and the sample. The *T*_2_ is inferred from the fit as the only free parameter.

The second sample consists of Si nanodiscs with a diameter of 200 nm, disc height of 150 nm and pitch 550 nm on a quartz substrate (see inset at Fig. 4a) covered by a polydimethylsiloxane (PDMS) layer for refractive index matching. The sample exhibits a strong narrow resonant dip in transmission at 818 nm. This dip corresponds to a mode inside the array excited due to the interaction of magnetic resonances in the single nanoparticles coupled through a diffraction order propagating along the array^41–45^. From the measured spectrum, we obtain the detuning from the central frequency of the biphoton field (Ω = 4.4 nm) and the optical thickness of the sample (*αL* = 4). It is important to note that since this sample consists of a single layer of resonant nanoparticles and its transmission is determined by the resonant interactions of the nanoparticles within the array, the optical thickness of the sample cannot be further reduced without losing its resonant properties. Thus this case corresponds to a situation when conventional transmission spectroscopy cannot be adequately applied for identification of *T*_2_ while the proposed methodology can successfully accomplish this task.

### Experimental setup

We produce photon pairs in a 0.8 mm long BBO crystal (Dayoptics) cut for type-I SPDC $(e\to oo)$ and pumped by a cw-laser at 407 nm (PhoxX 405–60 Omicron), see Fig. 1b. The pump laser power is set at 60 mW and the beam diameter is of 1.4 mm. Photons pairs are produced in a frequency degenerate non-collinear regime with emission angles *θ* = ±3 deg with respect to the pump beam. The photons are coupled into two single mode fibers (SMF) with fiber paddles in both arms used for polarization control. The SPDC spectrum has a bandwidth of 22 nm, as it is defined by the phasematching conditions and coupling to SMFs^46^. Additionally, we use an auxiliary laser (at 814 nm) which facilitates alignment of the interferometer (not shown). The beams at the input of the HOM interferometer have the same polarization. The half wave plate (HWP1) set at 45 deg is placed in one of the arms, and the beams are recombined on a polarizing beamsplitter (PBS1). In one arm of the interferometer we introduce a delay line using a motorized translation stage (Owis) with a translation step of 0.5 µm. In another arm we place a telescopic 1:1 imaging system, consisting of two lenses with *f* = 50 mm, which focuses the beam onto the sample (a spot size at the sample is 16 µm). A light from a halogen lamp (HL) is fed into the imaging system with a dichroic mirror (DM) and the image of the sample is captured by a CCD camera (Thorlabs). The beam combined at PBS1 passes through a half-wave plate (HWP2) set at 22.5 deg, which rotates the polarization by 45 deg and makes the photons indistinguishable. The photons are then split at a polarizing beamsplitter (PBS2) and coupled into two single mode fibers. We use an interference bandpass filter (IF; FF01-820/12-25, Semrock) tilted by 9 deg to tune its central transmission wavelength to 815 nm with FWHM = 21 nm. In the setup there is a possibility either (1) to connect the output of the fibers to a home-build grating spectrometer (resolution of 0.2 nm) for the spectral measurements, or (2) directly to single photon avalanche photodetectors (D1, D2; SPCM-AQR-14FC, Perkin Emler) for HOM dip measurements. Signals from the detectors are sent to a coincidence counting scheme (Ortec, TAC 556) with a time window of 3 ns. Typical acquisition time for each sample was about 10–15 min, depending on the sample transmission. The obtained results are then fitted with the theory using Matlab and Origin software.

## Results and Discussion

### Numerical considerations

**Figure 5:**
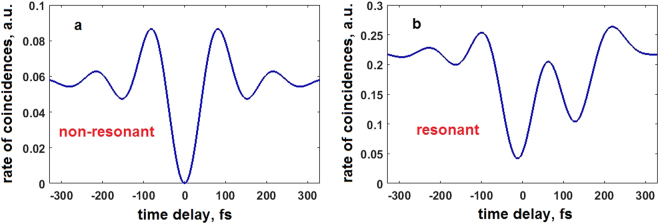
Numerical simulations. (**a**) Results for a non-resonant medium modeled by the Gaussian function with σ = 2 THz and *Ω* = 2 THz, the biphoton field is centered at 815 nm. (**b**) Results for a resonant medium modeled by the Lorentzian function with *αL* = 4, *T*_2_ = 475 fs, and Ω = 2 THz, the biphoton field is centered at 815 nm. The two plots are distinctively different as the dip becomes asymmetric and elongated for the case of the resonant media.

Here we demonstrate the difference between results obtained for non-resonant and resonant medium placed into the HOM interferometer. We consider the spectral amplitude of the biphoton field *F*(*v*) as described above in Methods, with both the filter function and the SPDC spectrum centered at 815 nm. First, we consider the situation when a non-resonant medium is placed into the interferometer and acts as a notch-filter. We model the transmission profile of the non-resonant medium with a Gaussian function:

$$H(\nu )=1-\frac{1}{{({\sigma }^{2}2\pi )}^{0.25}}\exp [-\frac{{(\nu -{\rm{\Omega }})}^{2}}{4{\sigma }^{2}}],$$

where σ is the parameter of the width linked with FWHM as ${\rm{\Delta }}{\nu }_{1/2}=2\sqrt{2\,\mathrm{ln}\,2}\sigma$, ν stands for the frequency detuning from the central wavelength, and *Ω* is the frequency detuning of the center of the transmission profile for the non-resonant medium from the center of the biphoton field. In our calculations we consider the following parameters σ = 2 THz (≈4.5 nm), and *Ω* = 2 THz. We substitute the parameters for the modeled medium and the biphoton field into Eq. 7 and calculate the shape of the HOM dip (Fig. 5a). Our calculations show, that the dip keeps its symmetry and it has a visibility equals to unity.

Next, we simulate the case of the resonant medium. It is modeled by a 2-level system with a Lorentzian shape of the transfer function with the following parameters: *αL* = 4, *T*_2_ = 475 fs, and Ω = 2 THz, see Eq. 8. The parameters of the incident biphoton field remain the same as in the previous case. The results of calculations using Eq. 7 are presented in Fig. 5b. It is clearly seen, that unlike in the previous case, the HOM dip takes an elongated and asymmetric shape. This confirms that the shape of HOM dip reflects the phase acquired by the photon due to the coherent response of the resonant medium.

### Experimental results

First we measure the HOM dip without any sample, see Fig. 3b. Our results show good quality of the entanglement with the uncorrected interference visibility of 92 ± 0.3%. The experimental results in Fig. 2b are fitted by Eq. 7, assuming that in the absence of a sample *H* = 1, and with the parameters of the biphoton field described in Methods. The fit yields a coefficient of determination R^2^ = 0.988. Subsequently, parameters of the biphoton field are used in the fitting of the experiments with the samples.

Then, we perform an experiment with the Nd:YAG crystal. We measure the transmission spectrum of the crystal using the spectrometer, and find that there are five absorption lines within the biphoton spectrum, see Fig. 3a. From the obtained spectrum we determine the detuning of each line from the central wavelength of the biphoton field and the sample’s optical thickness. Then, we measure the HOM dip with the Nd:YAG crystal in one of the arms of the interferometer. We find that the dip takes an asymmetric and elongated shape, see Fig. 3b. This is attributed to a coherent resonant response of the medium^39, 40^. Then we fit the interference pattern with Eqs 7–9 with the parameters defined from the spectrum and infer *T*_2_ for each line. The best fit yields R^2^ = 0.933 with the following values *T*_2_^*I*^ = 620 ± 50 fs, *T*_2_^*II*^ = 660 ± 50 fs, *T*_2_^*III*^ = 415 ± 30 fs, *T*_2_^*IV*^ = 710 ± 60 fs, *T*_2_^*V*^ = 215 ± 20 fs, where roman numerals denote corresponding resonant lines. We highlight that strong absorption in the sample results in broadening and overlap of spectral lines. In this case, dephasing times cannot be directly estimated from the spectral linewidths.

To ensure the validity of our method, we compare our results for the line IV (*αL* ^*I*V^ = 7.2) with spectroscopic data obtained for an optically thin Nd:YAG sample with similar Nd concentration^47^. This line has the strongest absorption among others, and it is used for pumping of solid-state lasers. Note, that for optically thin samples (*αL* < 1), the coherence time and width of the resonance are related as ${T}_{2}=1/\pi {\rm{\Delta }}{\nu }_{1/2}$ ^1^. Based on the literature data, for an optically thin sample the line IV has the FWHM of about 1 nm, which yields *T*_2_^*IV*^_thin sample_ ≈ 700 fs^47^. This value is consistent with our measurements (*T*_2_^*IV*^ = 710 ± 60 fs), thus ensuring the validity of our technique. It was also shown that for a number of solids at room temperature, for example dielectric crystals doped with rare-earth ions like Nd:YAG crystal, ${{T}_{2}}^{^{\prime} }$ dominates *T*_2_^*^, and correspondingly *T*_2_ ~ ${{T}_{2}}^{^{\prime} }$ describes a homogeneously broadened transition^48, 49^.

Next, we apply the developed methodology to the case when the optical thickness of the sample cannot be reduced without significant modification of its properties. We perform the experiment with an array of silicon nanodiscs with diffractively coupled magnetic dipole resonances (see Methods). Following the procedure described above, we first measure the transmission profile of the sample, which possesses a single dip at 818 nm, see Fig. 4a. We then measure the HOM dip, which has elongated and asymmetric shape, see Fig. 4b. We fit the obtained results using Eqs 7–9 with *T*_2_ as a single fitting parameter. The fit yields *T*_2_ = 130 ± 15 fs with R^2^ = 0.971. The measured *T*_2_ is attributed to a dephasing time between the coupled magnetic dipole moments inside neighboring Si nanoparticles constituting the resonant mode. It is analogous to the dephasing time of surface plasmon polaritons in metal nanostructures and nanoarrays, which has been measured earlier using conventional ultrafast spectroscopy techniques^50–52^. It is important to note, that dephasing in these systems is attributed to the collective excitation when an array works as a whole, and thus inhomogeneous and homogeneous broadening coincide^50^. Considering a measured value of the FWHM of 12 nm from Fig. 4a, we obtain *T*_2_ ≈ 70 fs, which is almost two times smaller rather than our experimental result. This discrepancy occurs due to the optical thickness of the sample being larger than unity (*αL* = 4), which broadens the linewidth and makes the direct calculation of *T*_2_ from the absorption spectra inadequate.

The temporal resolution of our technique is defined by the width of the spectrum of the biphoton field. In our experiment, it is 20 nm, which corresponds to a resolution of about 35 fs. It is on a par with existing high-performance femtosecond laser setups. With readily available methods for generation of broadband biphoton fields, it is feasible to achieve the temporal resolution down to a few femtoseconds or even less^53–55^. For example, SPDC generated in chirped crystals^53^ yields the spectral width of 300 nm, which corresponds to the temporal resolution of 7 fs. Moreover, high tunability of SPDC source allows to cover different spectral ranges by selecting different wavelength of a pump laser. Both, arbitrary shapes of resonant lines and spectral shape of a biphoton field, can be always accounted in numerical calculations see Eq. 7.

## Conclusion

In conclusion, we have demonstrated a new technique for measuring the dephasing time of a matter on the femtosecond time scale. Our approach utilizes the effect of the quantum two-photon interference of entangled photons. We showed that in the case of a resonant medium the shape of two-photon interference is determined by not only spectral transmission but also by a phase acquired through interaction with a medium. The technique uses entangled photons which are produced by a cw-laser, thus eliminating the need for complex and expensive femtosecond laser setups. It allows the measurement of dephasing times in optically thick samples, for which application of transmission spectroscopy is limited. Moreover, our approach does not suffer from the even orders of group velocity dispersion, which is one of the limiting factors in conventional methods. The technique operates at a single photon level and it can be useful for measurements of fragile biological, chemical and nanostructured samples. We believe that the technique will contribute to further development of ultrafast time-resolved spectroscopy in material science, biology, and chemistry.

## Electronic supplementary material

Supplementary Material
