# Supplementary material for: Quantum interference in the presence of a resonant medium
Source: Sci Rep. 2017 Sep 13;7:11444. doi: 10.1038/s41598-017-11694-z (PMC5597610; doi:10.1038/s41598-017-11694-z)
Supplement: Supplementary file 1 — Supplementary Material [file 41598_2017_11694_MOESM1_ESM.pdf]

Supplementary Information

Quantum interference in the presence of a resonant medium

Dmitry A. Kalashnikov, Elizaveta V. Melik-Gaykazyan, Alexey A. Kalachev, Ye Feng Yu,

Arseniy I. Kuznetsov, and Leonid A. Krivitsky

Let us consider a two-level resonant medium with a single Loretzian absorption line and assume that its central frequency coincides with the central frequency of the biphoton field. In this case, Eq. (8) takes the form

$$H(\omega_0+\nu)=\exp\left[-\frac{ib}{\nu+i/T_2}\right]\equiv H_b(\nu).$$

By performing the Fourier transformation, we obtain the following impulse-response function of the resonant medium:

$$H_b(t)=\frac{1}{2\pi}\int d\nu H_b(\nu)e^{-i\nu t}=\delta(t)-b\frac{J_1\left(2\sqrt{bt}\right)}{\sqrt{bt}}\theta(t)e^{-\frac{t}{T_2}}\equiv\delta(t)-\Phi_b(t),$$

where $J_1(x)$ is the Bessel function of the first kind, and $\theta(x)$ is the step function that is equal to 0 for $x<0$, 1 for $x>0$, and 1/2 if $x=0$.

To be more specific, consider the Gaussian spectral amplitude of the biphoton field:

$$|F(\nu)|^2=\frac{1}{\sigma\sqrt{2\pi}}\exp\left[-\frac{\nu^2}{2\sigma^2}\right]$$

so that its autocorrelation function reads

$$S(t)\equiv\frac{1}{2\pi}\int d\nu|F(\nu)|^2e^{-i\nu t}=\frac{1}{2\pi}\exp\left[\frac{t^2\sigma^2}{2}\right].$$

The coherence time and spectral FWHM of the biphoton field are $\tau_{coh}=2\sqrt{\ln 2}/\sigma$ and $\Delta\nu=2\sqrt{2\ln 2}\sigma$, respectively.

Since $H_b(\nu)=H_b^*(-\nu)$, from Eq. (8) we obtain:

$$P_c(\tau)=\frac{1}{2}\left[P_0-2\pi\int dtS(t)H_{2b}(2\tau-t)\right]=\frac{1}{2}\left[P_0-2\pi S(2\tau)+2\pi\int dtS(t)\Phi_{2b}(2\tau-t)\right],$$

Where

$$P_0=\int d\nu|F(\nu)H_b(\pm\nu)|^2.$$

Thus, the shape of the HOM dip, which is described by the function $P_c(\tau)-P_c(\infty)$, contains information about the impulse response function of the resonant medium through its convolution with the autocorrelation function of the biphoton field.

If the field coherence time is short (broadband field), the HOM dip becomes a function of the impulse response function only. Indeed, in this case we have

$$P_0\approx 1-2\pi|F(0)|^2b\left[I_0(bT_2)e^{-bT_2}+I_1(bT_2)e^{-bT_2}\right],$$

where $I_n(x)$ is the modified Bessel function of the first kind, and

$$2\pi\int dtS(t)\Phi_{2b}(2\tau-t)\approx 2\pi|F(0)|^2\Phi_{2b}(2\tau).$$

Finally, let us consider the case of broadband field and optical thin medium when $\tau_{coh} << T_2$ and $\alpha L << 1$. Taking into account that $|F(0)|^2 = \left(\sqrt{2\pi}\sigma\right)^{-1}$, we obtain

$$P_0 \approx 1 - \frac{\sqrt{2\pi}\alpha L}{2\sigma T_2},$$

$$\Phi_{2b}(2\tau) \approx \frac{\alpha L}{T_2}\theta(2\tau)e^{-\frac{2\tau}{T_2}},$$

so that

$$P_2(\tau) = \frac{1}{2}\left[1 - \exp\left(-\frac{\sigma^2(2\tau)^2}{2}\right) + \frac{\sqrt{2\pi}\alpha L}{2\sigma T_2}\left\{\theta(2\tau)e^{-2\tau/T_2} - \frac{1}{2}\right\}\right].$$

The first two terms in the square brackets describe the HOM dip that is observed without a resonant medium. The third term describes contribution of the optically thin resonant medium. It can be seen that the HOM dip becomes asymmetrical. For sufficiently large delay times $(\tau > 3\tau_{coh})$, the coincidence counting rate exponentially approaches $P_c(\infty) \approx 1/2\left[1 - 0.75\alpha L(\tau_{coh}/T_2)\right]$, which is slightly less than the value 1/2 observed without the resonant medium.
